# Videobasierte Psychotherapie: Aktuelle Rahmenbedingungen und Entwicklungen sowie Empfehlungen für die praktische Umsetzung

**DOI:** 10.1007/s00103-022-03644-6

**Published:** 2023-01-17

**Authors:** Lena Sophia Steubl, Harald Baumeister

**Affiliations:** Abteilung Klinische Psychologie und Psychotherapie, Institut für Psychologie und Pädagogik, Universität Ulm, Lise-Meitner-Str. 16, 89081 Ulm, Deutschland

**Keywords:** Digitalisierung, COVID-19, Ambulante Psychotherapie, Online, Praxis, Digitalization, COVID-19, Outpatient psychotherapy, Online, Practice

## Abstract

Videobasierte Psychotherapie ist ein möglicher digitaler Ansatz zur Bereitstellung psychotherapeutischer Behandlung – insbesondere dann, wenn Psychotherapie aufgrund von strukturellen Hindernissen nicht im unmittelbaren Kontakt stattfinden kann, zum Beispiel in Zusammenhang mit der COVID-19-Pandemie. Die Nutzung dieses Ansatzes hat sich sowohl als wirksam als auch als akzeptiert unter Psychotherapeutinnen und -therapeuten sowie Patientinnen und Patienten erwiesen. Rahmenbedingungen für die videobasierte Psychotherapie innerhalb der gesetzlichen Krankenversicherung in Deutschland und Empfehlungen für die praktische Umsetzung werden in diesem Artikel zusammengefasst.

## Abstract (en)

Video-based psychotherapy is a digital way to deliver psychotherapeutic treatment when traditional on-site psychotherapy cannot be provided due to structural barriers, such as those related to the COVID-19 pandemic. The use of this digital approach has proven to be both effective and accepted among therapists and patients. Legal frameworks for German therapists and recommendations for practical implementation are summarized in this article.

## Einleitung

In Deutschland werden etwa 25–30 % der psychisch Erkrankten fachspezifisch versorgt – psychotherapeutisch behandelt werden trotz nachgewiesener langfristiger Wirksamkeit und Empfehlung bei vielen Erkrankungen in den Leitlinien nur etwa 10–15 % [1]. Gründe hierfür liegen zum einen in einstellungsbezogenen Hindernissen wie der Stigmatisierung von psychischen Erkrankungen und deren Behandlung [2, 3]. Zum anderen spielen strukturelle Barrieren wie die Verfügbarkeit von evidenzbasierter Psychotherapie insgesamt und vor allem in ländlichen und abgelegenen Regionen eine Rolle [2, 3]. Insbesondere die strukturellen Barrieren hinsichtlich des Zugangs zu psychotherapeutischer Versorgung wurden durch die COVID-19-Pandemie und damit einhergehende Kontaktbeschränkungen verstärkt.

Einen Ansatz zur Überwindung dieser Herausforderung stellen digitale Interventionen dar, denn der allgegenwärtige digitale Wandel eröffnet auch für die Behandlung von Patientinnen und Patienten eine Vielzahl von neuen Möglichkeiten. Eine dieser neuen Möglichkeiten ist die videobasierte Psychotherapie, die durch die COVID-19-Pandemie sehr stark an Aufmerksamkeit und unmittelbarer Praxiserprobung gewonnen hat.

In diesem Beitrag sollen Hintergründe zu Bedarf, Wirksamkeit und Akzeptanz zusammengefasst sowie ein Überblick über die rechtlichen Rahmenbedingungen und hilfreiche Hinweise für die praktische Umsetzung gegeben werden.

## Digitale Ansätze in der Psychotherapie

Digitale Ansätze in der psychotherapeutischen Versorgung umfassen eine große Bandbreite an verschiedenen Darbietungsmöglichkeiten von Behandlungsbestandteilen, deren Wirksamkeit vielversprechend ist [4, 5]. So können psychotherapeutische Inhalte beispielsweise in begleiteten oder unbegleiteten Selbsthilfe-Interventionen – zertifiziert zum Verschreiben auf Rezept digitale Gesundheitsanwendungen (DiGA) genannt – mittels Smartphone-Apps oder verfügbar über einen Web-Browser dargeboten werden [5, 6]. Diese digitalen Interventionen sind häufig unter dem Begriff Internet- und Mobile-basierte Interventionen – kurz IMIs – zusammengefasst. „Begleitet“ bedeutet dabei, dass geschulte Personen, sogenannte E‑Coaches, Patientinnen und Patienten Unterstützung und Feedback synchron oder asynchron schriftlich oder verbal geben [7]. Die E‑Coaches unterscheiden sich dabei von IMI zu IMI in ihrer Qualifikation und auch eine rein automatisierte Unterstützung zum Beispiel in Form von Chatbots ist denkbar. Diese Form der digitalen Behandlung ist – wenn auch bisher vor allem im allgemeinmedizinischen Bereich verschrieben – durch die bestehenden Rahmenbedingungen mittlerweile etablierter Bestandteil unseres Versorgungsalltags in Deutschland [8]. Allerdings können die IMIs nicht nur als für sich stehende Selbsthilfe-Interventionen genutzt werden, sondern auch verzahnt mit der etablierten Behandlung, wie z. B. der Psychotherapie, im unmittelbaren Kontakt kombiniert oder auch konsekutiv aufeinander aufbauend angeboten werden [9]. So können Ressourcen geschont, die Dosis erhöht, eine schnellere Bereitstellung von Hilfe gewährleistet oder der Therapieprozess flexibilisiert werden [9]. Dieses Konzept der sogenannten verzahnten Psychotherapie (engl.: *Blended Therapy*) steht noch am Anfang der wissenschaftlichen Untersuchung [9–11].

Videobasierte Psychotherapie bildet ebenfalls eine in unserem Versorgungsalltag etablierte Unterform der digitalen Ansätze. Unter videobasierter Psychotherapie versteht man die psychotherapeutische Behandlung mittels Video im Sinne eines synchronen Austausches einer Psychotherapeutin oder eines Psychotherapeuten mit einer Patientin oder einem Patienten – oder mit mehreren in einem Gruppensetting – mit Hilfe elektronischer Datenverarbeitung zur Bild- und Tonübertragung [12]. Im Gegensatz zu den IMIs leistet die videobasierte Psychotherapie nicht unbedingt einen zusätzlichen Versorgungsbeitrag, da sie eher mit der üblichen Form der Psychotherapie, die im unmittelbaren Kontakt stattfindet, vergleichbar ist. Zwar können Zugangsprobleme zur Psychotherapie etwa durch eine verstärkte Barrierefreiheit vermindert und möglicherweise auch eine bessere Kontinuität der Behandlung ermöglicht werden (z. B. bei Umzug, einem weiten Anfahrtsweg oder körperlichen Erkrankungen), am Kontingent zur Verfügung stehender Psychotherapieangebote ändert sich hierdurch allerdings nichts. Die Stärke der videobasierten Psychotherapie liegt vielmehr darin, das Angebot der seit Jahrzehnten etablierten und evidenzbasierten Psychotherapie derart zu erweitern, dass sie auch Menschen erreicht, die ansonsten möglicherweise psychotherapeutisch unversorgt blieben.

## Wirksamkeit der videobasierten Psychotherapie

Die Wirksamkeit videobasierter Psychotherapie kann für eine Vielzahl von psychischen Erkrankungen als belegt gelten [13, 14]. Effektstärken für videobasierte Therapie werden mit mittleren bis hohen Werten (g = 0,54–1,34) angegeben [15]. Auch verglichen mit Psychotherapie im unmittelbaren Kontakt zeigen sich vielversprechende Ergebnisse. Zum Beispiel zeigt eine Metaanalyse von Norwood und Kollegen aus dem Jahr 2018 keine signifikante Unterlegenheit der videobasierten Psychotherapie gegenüber der Psychotherapie im unmittelbaren Kontakt hinsichtlich der Wirksamkeit in Bezug auf die Symptomreduktion [16]. Allerdings konnten in die Metaanalyse nur 4 randomisiert kontrollierte Studien eingeschlossen werden. 2 aktuellere Metaanalysen aus dem Jahr 2021 mit 36 beziehungsweise 43 eingeschlossenen Studien konnten allerdings ebenfalls zeigen, dass videobasierte Psychotherapie statistisch nicht weniger wirksam ist als Psychotherapie im unmittelbaren Kontakt, auch wenn hier zum Teil nichtrandomisierte Studien eingeschlossen wurden [15, 17]. Auch in Hinblick auf die Nutzung zu diagnostischen Zwecken zeigten sich keine statistisch signifikanten Unterschiede zwischen einer psychodiagnostischen Erhebung videobasiert oder im unmittelbaren Kontakt [17].

Insgesamt ist allerdings einschränkend zu betrachten, dass aufgrund der in den Studien verwendeten und beschriebenen sehr unterschiedlichen digitalen Ausstattung bzw. Vorgehensweisen die Generalisierung der Ergebnisse erschwert wird [15–18]. Außerdem fehlen in den Beschreibungen der Studiencharakteristika häufig genauere Angaben sowohl zu den Vergleichsgruppen als auch zur genauen Ausgestaltung der videobasierten Psychotherapie, sodass eine über das jeweilige Behandlungsformat hinausgehende Äquivalenz der therapeutischen Behandlung innerhalb der Gruppen nicht als gesichert gelten kann [17]. In Hinblick auf die behandelte psychische Erkrankung zielten die meisten eingeschlossenen Studien darüber hinaus vorrangig auf Angststörungen, Depressionen und posttraumatische Belastungsstörung ab und nutzten vor allem kognitiv-verhaltenstherapeutische Ansätze [15, 17]. Die vorrangige Nutzung kognitiv-verhaltenstherapeutischer Ansätze in der videobasierten Psychotherapie könnte mit der standardisierten und manualisierten Natur des Psychotherapieverfahrens zusammenhängen sowie mit deren geringerer Abhängigkeit von Beziehungsdynamiken in der Behandlungssituation [15]. Bisher noch nicht ausreichend untersucht sind neben Fragen zur differentiellen Wirksamkeit, zur Kosteneffektivität und zu Nebenwirkungen damit aber auch potenzielle moderierende Effekte von Störungsbildern und Therapieverfahren [13].

## Akzeptanz der videobasierten Psychotherapie und Nutzung in der Praxis

Neben der belegten Wirksamkeit spielt selbstverständlich auch die Akzeptanz der videobasierten Psychotherapie eine große Rolle, da sie sowohl vonseiten der Psychotherapeutinnen und -therapeuten als auch vonseiten der Patientinnen und Patienten letztlich ausschlaggebend für die tatsächliche Inanspruchnahme der Therapiemöglichkeit ist. Insgesamt bewerten sowohl Psychotherapeutinnen und Psychotherapeuten als auch Patientinnen und Patienten die videobasierte Psychotherapie dabei als gut durchführbar und die damit einhergehenden Vorteile (v. a. Flexibilität) werden erkannt [19, 20].

In diesem Zusammenhang wird außerdem die therapeutische Beziehung als wichtiger Wirkfaktor der Psychotherapie im unmittelbaren Kontakt auch in der videobasierten Psychotherapie tendenziell als gut bewertet – und das auch bei niedrigen Internet-Bandbreiten und schlechter Übertragungsqualität [3]. Tatsächlich gibt es sogar Hinweise darauf, dass sich Patientinnen und Patienten in videobasierter Psychotherapie mehr beteiligen und sicherer fühlen [3]. Zusätzlich können die teils notwendigen zusätzlichen Vorbereitungen und Bemühungen von Psychotherapeutinnen und -therapeuten (z. B. bei der Auseinandersetzung mit den technischen Möglichkeiten zur Umsetzung von bestimmten Interventionsinhalten oder in Hinblick auf detailliertere Nachfragen zu Gestik und Mimik) zumindest am Anfang einer videobasierten psychotherapeutischen Behandlung zu verbesserten Ergebnissen beitragen [3].

Allerdings zeigen Studien auch, dass insbesondere Psychotherapeutinnen und -therapeuten noch Vorbehalte äußern und die therapeutische Beziehung in der videobasierten Psychotherapie schlechter bewerten als ihre Patientinnen und Patienten [21]. In einer Umfrage der Bundespsychotherapeutenkammer zur Nutzung von videobasierter Psychotherapie während der ersten Welle der COVID-19-Pandemie gaben zum Beispiel befragte Psychotherapeutinnen und -therapeuten vor allem Schwierigkeiten hinsichtlich instabiler Internetverbindungen, fehlender technischer Ausstattung, fehlender ungestörter Räumlichkeiten bei Patientinnen und Patienten zu Hause und mangelnder Durchführbarkeit von bestimmten Interventionen (z. B. traumatherapeutische Interventionen wie EMDR^1^, erlebnisorientierte Techniken) an [19]. Zudem gibt es Evidenz dafür, dass die Akzeptanz videobasierter Psychotherapie insbesondere bei älteren Patientinnen und Patienten geringer ausfällt [22].

Hinsichtlich der tatsächlichen Nutzung videobasierter Psychotherapie hat die Bundespsychotherapeutenkammer 2020 während der ersten Welle der COVID-19-Pandemie 3500 Psychotherapeutinnen und -therapeuten zu ihren Erfahrungen befragt [19]. Dabei zeigte sich, dass etwa 9 von 10 Psychotherapeutinnen und Psychotherapeuten Behandlungen mittels Video durchgeführt haben – die Mehrheit davon zum ersten Mal im Laufe der COVID-19-Pandemie [19]. Ebenfalls etwa 9 von 10 befragten Psychotherapeutinnen und -therapeuten können sich vorstellen auch nach dem Ende der COVID-19-Pandemie weiterhin Behandlungen mittels Video durchzuführen, etwa die Hälfte aber nicht mehr so häufig wie während der COVID-19-Pandemie [19].

## Rahmenbedingungen innerhalb der gesetzlichen Krankenversicherung in Deutschland

Die rechtlichen Rahmenbedingungen für die videobasierte Psychotherapie in Deutschland haben sich nicht zuletzt durch die COVID-19-Pandemie in den letzten Jahren stetig gewandelt. Seit dem 22. Deutschen Psychotherapeutentag am 17.11.2018 ist durch eine Anpassung des § 5 Musterberufsordnung (MBO) videobasierte Psychotherapie berufsrechtlich möglich und seit dem 01.04.2019 sind Psychotherapeutinnen und -therapeuten berechtigt, videobasierte Psychotherapie als Leistung der gesetzlichen Krankenversicherung durchzuführen [12]. Die Abrechnung ist seit dem 01.10.2019 möglich – mit Einschränkungen hinsichtlich der möglichen Anteile an der Gesamtheit der erbrachten Leistungen [12]. Die im Zuge der Corona-Schutzmaßnahmen getroffenen Sonderregelungen, die entsprechende Beschränkungen der Anteile aufhoben, liefen zum 31.03.2022 aus [23]. Zum 01.04.2022 wurde in der Folge der ursprünglich geltende zulässige Anteil von mittels Video erbrachten Leistungen von 20 % auf 30 % angepasst [23]. Dieser Höchstwert gilt für den Anteil von Patientinnen und Patienten, die mittels Video behandelt werden, sowie für den Anteil einer Leistung innerhalb eines Quartals, der mittels Video erbracht wird [12]. Zum 01.07.2022 gilt dieser Anteil für alle per Video möglichen Leistungen zusammen, die von einer Praxis je Quartal abgerechnet werden, und nicht mehr je einzelner Leistung, mit Ausnahme der Akutbehandlung [24]. Der Standard bleibt demnach weiterhin die Behandlung im persönlichen Kontakt.

Mittels Video durchgeführt und abgerechnet werden können: Akutbehandlungen, Richtlinienpsychotherapie, Gruppenpsychotherapien mit bis zu 8 Patientinnen und Patienten, gruppenpsychotherapeutische Grundversorgung, Rezidivprophylaxe, psychotherapeutische Gespräche, neuropsychologische Therapie, übende und suggestive Interventionen sowie standardisierte Testverfahren [12]. Dagegen müssen die psychotherapeutische Sprechstunde, probatorische Sitzungen, Hypnose und projektive Verfahren immer im unmittelbaren persönlichen Kontakt erfolgen. Psychometrische Testverfahren sind nur bei Erwachsenen mittels Video möglich. Für psychotherapeutische Gespräche und neuropsychologische Therapie ist vorab ein persönlicher Kontakt notwendig.

Die Behandlung mittels Video muss über einen zertifizierten Videodienstanbieter erfolgen, wobei zugelassene Anbieter über die Homepage der Kassenärztlichen Bundesvereinigung eingesehen werden können [12, 24]. Zudem muss die Behandlung auch in diesem Fall vonseiten der Psychotherapeutinnen und -therapeuten in den jeweiligen Praxisräumen stattfinden [12]. Ausnahmen können mit den jeweils berufs- und sozialrechtlich verantwortlichen Stellen besprochen werden [25]. In jedem Fall ist darauf zu achten, dass auch bei der videobasierten Psychotherapie eine vertrauliche und störungsfreie Kommunikation ermöglicht wird [12]. So ist auch hier eine regelhafte Behandlung vonseiten der Psychotherapeutinnen und -therapeuten vom Ausland oder von zu Hause aus nicht vorgesehen. Von der Patientin oder dem Patienten ist eine ausdrückliche Einwilligung zur Behandlung mittels Video nach einer ausführlichen Aufklärung einzuholen und die Patientin oder der Patient muss zu Beginn der Sitzung – bei Bedarf durch Vorzeigen der elektronischen Gesundheitskarte – identifiziert werden [12]. Bei Patientinnen und Patienten, die noch nicht 16 Jahre alt sind, ist für die Behandlung mittels Video eine Einwilligung der Sorgeberechtigten notwendig [12].

Bei der Behandlung mittels Video können die herkömmlichen Grundpauschalen sowie Zuschläge für die fachärztliche Grundversorgung abgerechnet werden, sofern zuvor bei der jeweiligen Kassenärztlichen Vereinigung angezeigt wurde, dass ein zertifizierter Videodienstanbieter genutzt wird [12]. Sollte eine Patientin oder ein Patient in einem Quartal nur mittels Video gesehen werden, so erfolgt ein Abschlag von 20 % auf die Grundpauschale und den Zuschlag für die fachärztliche Grundversorgung [12]. Zusätzlich zu den herkömmlichen Zuschlägen gibt es die Möglichkeit der Abrechnung eines Technikzuschlags und ebenfalls die eines Zuschlags für die Authentifizierung der Patientin oder des Patienten [12].

Insgesamt wird die durch die vorgegebenen Rahmenbedingungen verringerte Flexibilität im Einsatz videobasierter Psychotherapie von Psychotherapeutinnen und -therapeuten kritisiert [23]. Die hier dargestellten rechtlichen Rahmenbedingungen sollten allerdings als zeitlich begrenzt gültig verstanden werden, da sie einen Versorgungsbereich betreffen, der einem dynamischen Wandel unterliegt. Es sind jederzeit die aktuellen Vorgaben einzuhalten.

## Empfehlungen für die Umsetzung

Für die Umsetzung von videobasierter Psychotherapie in der Praxis gilt es, neben den allgemeinen rechtlichen Rahmenbedingungen noch eine Reihe weiterer Faktoren zu beachten, die von Psychotherapeutinnen und -therapeuten auch mit ihren Patientinnen und Patienten besprochen werden sollten. Die Interessengruppe E‑Health in der Fachgruppe Klinischen Psychologie und Psychotherapie der Deutschen Gesellschaft für Psychologie (DGPs) hat im Zusammenhang mit der COVID-19-Pandemie entsprechende Empfehlungen für die Vorbereitung und Durchführung von videobasierter Psychotherapie veröffentlicht [25].

Vor dem ersten Gespräch sollten demzufolge zum Beispiel sowohl Psychotherapeutinnen und -therapeuten als auch Patientinnen und Patienten für eine angemessene Umgebung sorgen. Es sollte darauf geachtet werden, dass die Behandlung in einem geschlossenen und ruhigen Raum durchgeführt und sichergestellt wird, dass die Sitzungen weder von anderen Personen (z. B. Familienmitgliedern, Mitbewohnerinnen und -bewohnern) noch von Geräten (z. B. Handy, Türklingel) oder Haustieren unterbrochen werden. Für die Psychotherapeutin oder den Psychotherapeuten müssen auch für videobasierte Psychotherapie die eigenen Praxisräume genutzt werden. Außerdem empfiehlt es sich insbesondere für Patientinnen und Patienten, trotz der privathäuslichen Umgebung auf angemessene Kleidung und einem angemessenen Ort für die Durchführung des Gesprächs zu achten (z. B. am Tisch sitzen und nicht im Bett liegen), um beiderseitige Wertschätzung der psychotherapeutischen Sitzung zu erhalten. Das Handy sollte für den Zeitraum beiseitegelegt werden.

Dringend notwendig ist eine gute und stabile Internetverbindung. Hier kann beispielsweise eine direkte Verbindung per Kabel helfen. Für den Fall eines plötzlichen Abbruchs der Verbindung – möglicherweise auch durch die Patientin oder den Patienten bewusst induziert – sollte vorher besprochen werden, wie in diesem Fall vorgegangen wird, wie die Patientin oder der Patient zu erreichen ist und wo sie oder er sich befindet. Zur Verbesserung der Tonqualität bietet es sich sowohl für Psychotherapeutinnen und -therapeuten als auch Patientinnen und Patienten an, ein Headset mit Mikrofon zu verwenden.

Materialien können zum Beispiel vor der Sitzung versandt werden oder während der Sitzung über einen geteilten Bildschirm gemeinsam betrachtet und bearbeitet werden. Insbesondere psychotherapeutische Inhalte, wie ausgefüllte Arbeitsblätter, sollten über gesicherte Plattformen wie das Videokonferenzsystem des zertifizierten Videodienstanbieters ausgetauscht werden. Als Ersatz für ein Flipchart bietet sich ein Blatt Papier an, das in die Kamera gehalten werden kann. Alternativ können technische Lösungen zum Beispiel des jeweiligen Videodienstanbieters genutzt werden.

Während des Gesprächs sollten von allen Beteiligten in jedem Fall sowohl Gesicht als auch Oberkörper gezeigt werden, sodass sowohl Mimik als auch Gestik übertragen werden. Da der für Videogespräche typische Blick auf das Bild des Gegenübers oder sich selbst distanziert wirken kann, sollte zumindest immer wieder auf einen bewussten Blick in die Kamera geachtet werden. Ebenfalls ist von beiden Seiten auf gute Lichtverhältnisse zu achten. Da es für manche Patientinnen und Patienten irritierend wirken kann das eigene Bild zu sehen, kann hier besprochen werden, ob die jeweilige Funktion des Videodienstanbieters abgeschaltet oder verdeckt werden kann. Insgesamt sollte die Patientin oder der Patient ermutigt werden nachzufragen, wenn es in der Kommunikation zu Unklarheiten kommt. Mögliche Einschränkungen sollten offen besprochen werden.

## Fazit

Videobasierte Psychotherapie ist eine wirksame und akzeptierte Möglichkeit zur barrierefreieren Bereitstellung psychotherapeutischer Hilfe – insbesondere dann, wenn eine Psychotherapie im unmittelbaren Kontakt (z. B. durch Umzug, einen weiten Anfahrtsweg oder körperliche Erkrankung) nicht möglich oder schwierig durchführbar ist. Auch die rechtlichen Rahmenbedingungen in Deutschland wurden mittlerweile so angepasst, dass eine psychotherapeutische Versorgung von Patientinnen und Patienten mittels Video zumindest anteilig möglich ist. Vonseiten der behandelnden Psychotherapeutinnen und -therapeuten ist es zum Teil notwendig, die Behandlung anzupassen und einige Rahmenbedingungen im Voraus mit Patientinnen und Patienten zu klären. Einschränkend ist in Hinblick auf die Überwindung von Versorgungsdefiziten zu betonen, dass die videobasierte Psychotherapie nicht zu einem Mehr an Versorgungsangebot führt. Diese Möglichkeit ergibt sich erst, wenn synchron dargebotene Psychotherapie – vor Ort oder videobasiert – mit asynchron vermittelten Selbsthilfeangeboten, wie z. B. digitalen Gesundheitsinterventionsangeboten, verzahnt wird [9, 26].

Zudem muss beachtet werden, dass Betroffene von den Angeboten ausgeschlossen sein können, wenn bestimmte Voraussetzungen nicht erfüllt sind (z. B. Internetverbindung, Räumlichkeit zur ungestörten Durchführung der Behandlung). Trotz dieser Einschränkungen kann videobasierte Psychotherapie einen bedeutsamen Mehrwert zur Versorgungsgerechtigkeit in Deutschland liefern, indem Psychotherapie für mehr Betroffene erreichbar wird.

## Footnotes

1 EMDR: *Eye Movement Desensitization and Reprocessing* (therapeutisches Verfahren, das regelmäßige Augenbewegungen vor allem zur Behandlung von posttraumatischer Belastungsstörung einsetzt).
